# Genome-wide association study of dilated cardiomyopathy-induced heart failure associated with renal insufficiency in a Chinese population

**DOI:** 10.1186/s12872-023-03370-0

**Published:** 2023-06-30

**Authors:** Yuexin Hu, Liangli Jin, Zhi Wang

**Affiliations:** 1 Department of Cardiovascular Medicine, Affiliated Nanjing Brain Hospital, Nanjing Medical University, No. 246 Guangzhou Road, Nanjing, Jiangsu, 210008 China; 2 Department of Cardiovascular Medicine, Nanjing Chest Hospital, Nanjing, China

**Keywords:** Heart failure, Renal insufficiency, Dilated cardiomyopathy, Single-nucleotide polymorphisms, Cardiorenal syndrome

## Abstract

**Background:**

As it is unclear whether there is genetic susceptibility to cardiorenal syndrome (CRS), we conducted a genome-wide association study of dilated cardiomyopathy (DCM)-induced heart failure (HF) associated with renal insufficiency (RI) in a Chinese population to identify putative susceptibility variants and culprit genes.

**Methods:**

A total of 99 Han Chinese patients with DCM-induced chronic HF were selected and divided into one of three groups, namely, HF with normal renal function (Group 1), HF with mild RI (Group 2) and HF with moderate to severe RI (Group 3). Genomic DNA was extracted from each subject for genotyping.

**Results:**

According to Gene Ontology (GO) function and Kyoto Encyclopedia of Genes and Genomes (KEGG) pathway analysis, top 10 lists of molecular function, cell composition and biological process of differential target genes and 15 signalling pathways were discriminated among the three groups. Additionally, sequencing results identified 26 significantly different single-nucleotide polymorphisms (SNPs) in the 15 signalling pathways, including three SNPs (rs57938337, rs6683225 and rs6692782) in ryanodine receptor 2 (*RYR2*) and two SNPs (rs12439006 and rs16958069) in *RYR3*. The genotype and allele frequencies of the five SNPs in *RYR2* and *RYR3* were significantly differential between HF (Group 1) and CRS (Group 2 + 3) patients.

**Conclusion:**

Twenty-six significantly different SNP loci in 17 genes of the 15 KEGG pathways were found in the three patient groups. Among these variants, rs57938337, rs6683225 and rs6692782 in *RYR2* and rs12439006 and rs16958069 in *RYR3* are associated with RI in Han Chinese patients with heart failure, suggesting that these variants may be used to identify patients susceptible to CRS in the future.

## Background

In the population, differences in nucleotide sequences among individuals are known as genetic polymorphisms. Among the types of genetic variations found in genes, mutations are a special, rare type of polymorphism, whereas single-nucleotide polymorphisms (SNPs), which are the replacement of a single base, are found extensively throughout the genome, having a frequent distribution and readily detectable via automation and batch detection. This abundance and availability is considered a new generation of genetic markers [1].

Relationships between disease-causing or -associated genetic polymorphisms and clinical phenotypic diversity have been a research focus for decades. Numerous studies have shown evidence of an association between SNPs and genetic susceptibility to various human diseases. More specifically, polymorphisms in genes have been associated with heart disease, including coronary heart disease, hypertension, cardiomyopathy and congenital heart disease [2–7].

Recently, interactions between the heart and kidneys have attracted attention. An epidemiological study showed that more than 50% of patients with chronic heart failure (HF) are complicated with renal insufficiency (RI), namely, cardiorenal syndrome (CRS) [8]. Further, other studies have shown that the deterioration of HF or the sympathetic nervous system (SNS), over-activation of the renin–angiotensin–aldosterone system (RAAS), drugs or other factors lead to cardiorenal syndrome [9]. Recently, Kao et al. performed a genome-wide association study to analyse whether SNPs were associated with HF with preserved ejection fraction (HFpEF). They found that 9 SNPs were associated with HFpEF, representing genes involved in myocyte proliferation, transforming growth factor-beta/erbB signalling and extracellular matrix formation [10]. The result from a cohort study on chronic kidney disease (CKD) in Saudi Arabian populations showed that rs4821480 in MYH9 was significantly associated with an increased risk of development of CKD [11]. No prior research has evaluated the association between SNPs and RI risk in Han Chinese with HF. The purpose of this genome-wide association study was to identify genetic variants associated with dilated cardiomyopathy (DCM)-induced HF and RI in a Chinese population.

## Materials and methods

### Subjects

In this non-interventional retrospective cohort study, consisting of 99 Han Chinese patients with chronic compensatory HF hospitalised in our hospital from January 2020 to August 2021. The diagnosis of HF was based on Chinese guidelines for the diagnosis and treatment of HF [12] and ESC guidelines for the diagnosis and treatment of acute and chronic HF [13]. All patients have typical clinical symptoms and signs of HF and a clear history of DCM, which was diagnosed according to the guideline protocol from the British Society of Echocardiography and Chinese Society of Cardiology. The clinical diagnostic criteria of DCM are objective evidence of left ventricular dilatation (> 112% corrected for age and body surface area and/or left ventricular end-diastolic diameter [LVDd] > 5.0 cm in females or LVDd > 5.5 cm in males) with reduced function (fractional shortening < 25% and/or left ventricular ejection fraction < 45%), excluding secondary causes, including hypertension, coronary artery disease, excess alcohol consumption, tachycardia-induced cardiomyopathy, systemic or pericardial disease, *cor pulmonale* and congenital heart disease [14, 15]. The exclusion criteria were pregnancy, diabetes, primary renal disease, primary liver disease, blood disease, tumour and metabolic and autoimmune diseases. All recruited subjects signed informed consent, and this study was approved by the ethics committee of Nanjing Brain Hospital (Approval ID: 2020-KY155-01, 2020-KY040-01).

### Cohort groups

The estimated glomerular filtration rate (eGFR) was calculated according to the modified MDRD formula for Chinese population (eGFR = 175 × [creatinine (mg/dL)]^−1.234^ × [age (years)]^−0.179^ × sex (male = 1, female = 0.79) [16, 17], and patients were divided into one of the three following groups: Group 1: 26 cases with normal renal function; Group 2: 40 cases with mild RI (60 ml/min · 1.73 m^2^ ≤ eGFR < 90 ml/min · 1.73 m^2^) and Group 3: 33 cases with moderate to severe RI (eGFR < 60 ml/min · 1.73 m^2^). General clinical data and laboratory examination data were collected and echocardiographic parameters were recorded of patients from all three groups. In order to control for bias, the selected patients with HF did not undergo any adjustment of the renin-angiotensin-aldosterone system (RAAS) antagonist dosage within the first month before enrolment, and the selected patients with HF had stable conditions and no deterioration of heart or renal function for at least 1 month before enrolment.

### Blood biochemistry

Fasting venous blood collection was performed in all subjects. High fat diet and drinking were prohibited 24 h before blood collection. Serum was obtained after centrifugation, and blood chemistry was determined using an Olympus automatic biochemical analyser. The serum N-terminal pro-brain natriuretic peptide (NT-proBNP) level was quantitatively detected using a Roche electrochemiluminescence immunoanalyser and the corresponding reagent, with a determination range of 5–35,000 pg/ml.

### Echocardiography

Subjects underwent cardiac colour Doppler examination (Hewlett-Packard). The left atrial diameter (LA), left ventricular end systolic diameter (LVS), left ventricular end diastolic diameter (LVD), ventricular septum and left ventricular posterior wall thickness were measured on the long axis of two chamber sections of the left ventricle. The left ventricular ejection fraction (LVEF) was measured by the Simpson method, and echocardiography results were independently assessed by an ultrasound physician. Each parameter was determined three times and the average value was taken.

### Genomic DNA extraction and quality control

Peripheral venous blood (3 ml) was collected in EDTA anticoagulant tubes from patients, and genomic DNA was extracted using a Whole-blood Genomic DNA Extraction kit (Tiangen Biochemical Technology). The DNA purity was determined using an ultraviolet spectrophotometer, with an OD 260/280 ratio between 1.7 and 2.1. DNA samples were stored at − 80 °C until use.

### Sequencing

For library generation, genomic DNA was randomly fragmented, followed by enzymatic end repair and adaptor ligation using DNA ligase. The library was further purified to remove redundant connectors and unconnected DNA fragments. The prepared library was added to a chip, followed by the addition of dNTPs and polymerase for complementary DNA strand synthesis. The DNA double strand is disrupted by the addition of a sodium hydroxide solution, and the strand not covalently bound to the chip is eluted. Next, a neutralisation solution is added, followed by the addition of polymerase and dNTPs to synthesise a new DNA strand. This process is repeated to obtain a cluster formed by single fragment cloning. After obtaining single strands for controllable sequencing, index sequencing was performed. The scanned image file and original data file generated by Illumina iSCAN were used for analysis to obtain the results of SNP genotyping, copy number variation (CNV) and CNV bookmark results list of cohort samples.

### Gene Ontology (GO) function and Kyoto Encyclopedia of genes and genomes (KEGG) pathway analysis

SNP locus information among the different groups was analysed to identify differential target SNP loci, and the obtained SNP information was then annotated to generate a gene list. Based on the involved underlying pathways and functions, the annotated gene list and DAVID (https://david.ncifcrf.gov/) were used for GO function analysis and KEGG pathway analysis. According to the screened gene list, the p-value representing whether the GO and KEGG function sets were significantly enriched in the differential target gene list was calculated using hypergeometric distribution testing, and the resulting p-value was corrected using Benjamin and Hochberg multiple testing to obtain the false discovery rate.

### Statistical analysis

IBM SPSS Statistics for Windows version 19 (IBM Corp., Armonk, NY, USA) was used for data analysis. Measurement data with normal distribution are expressed as mean ± standard error of the mean (SEM). Measurement data with non-normal distribution are expressed as median (Q1, Q3). Comparisons between different groups of subjects were statistically analysed by unpaired *t* test. The counting data were expressed as a percentage and analyzed using a chi-square test. P < 0.05 was considered statistically significant.

## Results

### Clinical baseline characteristics

Clinical baseline characteristics of our cohort are listed in Table 1. Compared with patients with HF and normal renal function (Group 1), we found that the mean age of patients with HF and mild (Group 2) or moderate to severe RI (Group 3) was increased, with the highest mean age found in patients from Group 3. In compliance with the guidelines for the treatment of HF and ethical requirements, all patients with HF received angiotensin-converting enzyme inhibitor/angiotensin receptor blocker (ACE-I/ARB), beta-blockers or Aldactone (ALD) without drug use contraindications. The application rates of beta-blocker, ACE-I/ARB and ALD were 84.6%, 76.9% and 84.6% in Group 1, 80%, 77.5% and 92.5% in Group 2, and 69.7%, 57.6% and 78.8% in Group 3, respectively. Statistical analysis showed no significant difference in the application rate of beta-blocker, ACE-I/ARB or ALD among the three groups. There were no significant differences in cardiac parameters (LVD, LVS and LVEF), blood lipid profile and troponin levels among the three groups. The NYHA grade in patients from Group 3 was significantly higher than those from Groups 1 and 2, and levels of CRP and NT-proBNP in patients from Group 3 were higher than those in Group 1. Further, compared with patients from Group 1, levels of renal function markers (BUN, Cr, CyC and eGFR) in patients from Groups 2 and 3 were significantly higher, with the highest levels found in patients from Group 3.

**Table 1:** Clinical baseline characteristics of three groups

	Group1 (n = 26)	Group2 (n = 40)	Group3 (n = 33)
Age(years)	58.04 ± 2.52	65.65 ± 2.4*	72.58 ± 2.2*#
Sex(male)	22(84.62%)	35(87.50%)	25(75.76%)
Medication Blocker (%) ACE-I/ARB (%) ALD (%)	22(84.6) 20(76.9) 22(84.6)	32(80) 31(77.5) 37(92.5)	23(69.7) 19(57.6) 26(78.8)
NYHA class	2.85 ± 0.14	3.10 ± 0.10	3.61 ± 0.16*#
Echocardiography LVDs (mm) LVDd (mm) LVEF(%)	51.04 ± 2.00 64.62 ± 1.81 42.96 ± 1.78	50.98 ± 1.48 65.60 ± 1.38 42.68 ± 1.14	52.03 ± 1.77 65.76 ± 1.68 41.91 ± 1.42
Laboratory			
CRP(mg/L)	8.07 ± 2.32	7.38 ± 1.62	16.1 ± 2.75*#
WBC (10^9/L)	6.97 ± 0.45	6.25 ± 0.30	6.72 ± 0.35
TC (mmol/L)	4.28 ± 0.19	4.45 ± 0.20	3.85 ± 0.20#
LDL-C (mmol/L)	2.58 ± 0.13	2.64 ± 0.14	2.18 ± 0.15#
HDL-C (mmol/L)	1.37 ± 0.10	1.36 ± 0.05	1.32 ± 0.07
Triglycerides (mmol/L)	1.22 ± 0.10	1.35 ± 0.15	1.06 ± 0.12
BUN (mmol/L)	6.03 ± 0.34	7.16 ± 0.31*	10.80 ± 0.95*#
Cr (µmol/L)	72.08 ± 2.93	95.73 ± 2.80*	143.3 ± 9.58*#
Cyc(mg/L)	0.85(0.71,0.96)	1.17(0.98,1.52)*	1.85(1.43,2.24)*#
eGFR (ml/min·1.73m2)	100.9(94.93,110.1)	71.35(66.03,79.93)*	45.90(40.85,54.45)*#
TNI(ng/mL)	0.01(0.01,0.05)	0.03(0.01,0.06)	0.02(0.01,0.05)
NT-proBNP (pg/mL)	3651 ± 1376	6252 ± 1067	9159 ± 1437*

**Abbreviations**: ACE-I, Angiotensin converting enzyme inhibitor; ARB, angiotensin receptor blocker; ALD, aldactone; NYHA, New York Heart Association;LVDs, left ventricular end systolic diameter; LVDd, left ventricular end diastolic diameter; LVEF, left ventricular ejection fraction; CRP, C reaction protein; WBC, white blood cell; TC, total cholesterol; LDL-C, low density lipoprotein-cholesterol; HDL-C, high density lipoprotein-cholesterol; BUN, blood urea nitrogen; Cr, creatinine; Cyc, Cystatin C; eGFR, estimated glomerular filtration rate; TNI, Troponin I; NT-proBNP, N-terminal pro-brain natriuretic peptide. Data are presented as mean ± SEM. *p < 0.05, vs.group1; #p < 0.05, vs.group2

### GO enrichment analysis

**Fig. 1:**
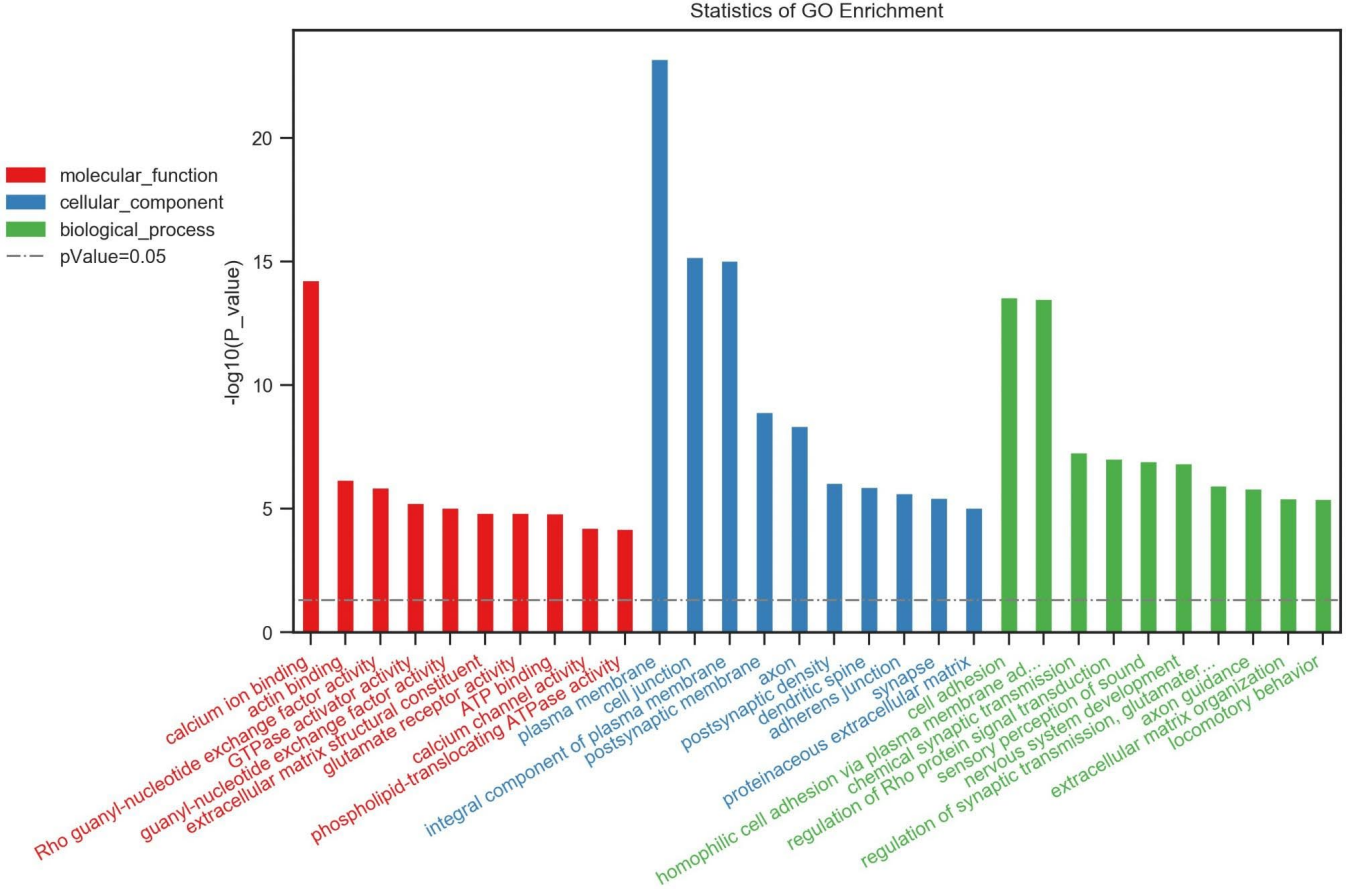
Statistics of Gene Ontology (GO) enrichment between Groups 1 and 2

**Fig. 2:**
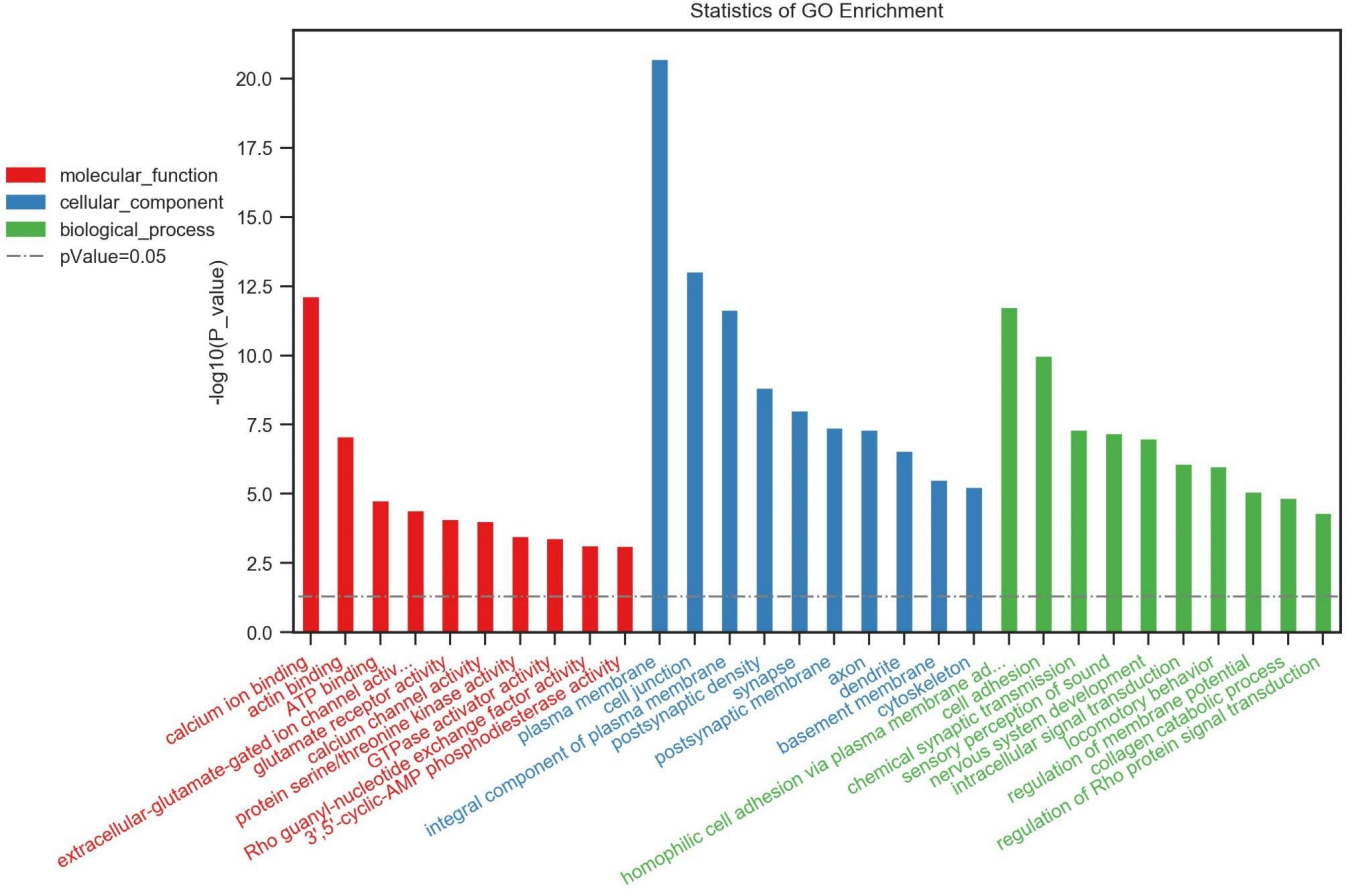
Statistics of Gene Ontology (GO) enrichment between Groups 2 and 3

**Fig. 3:**
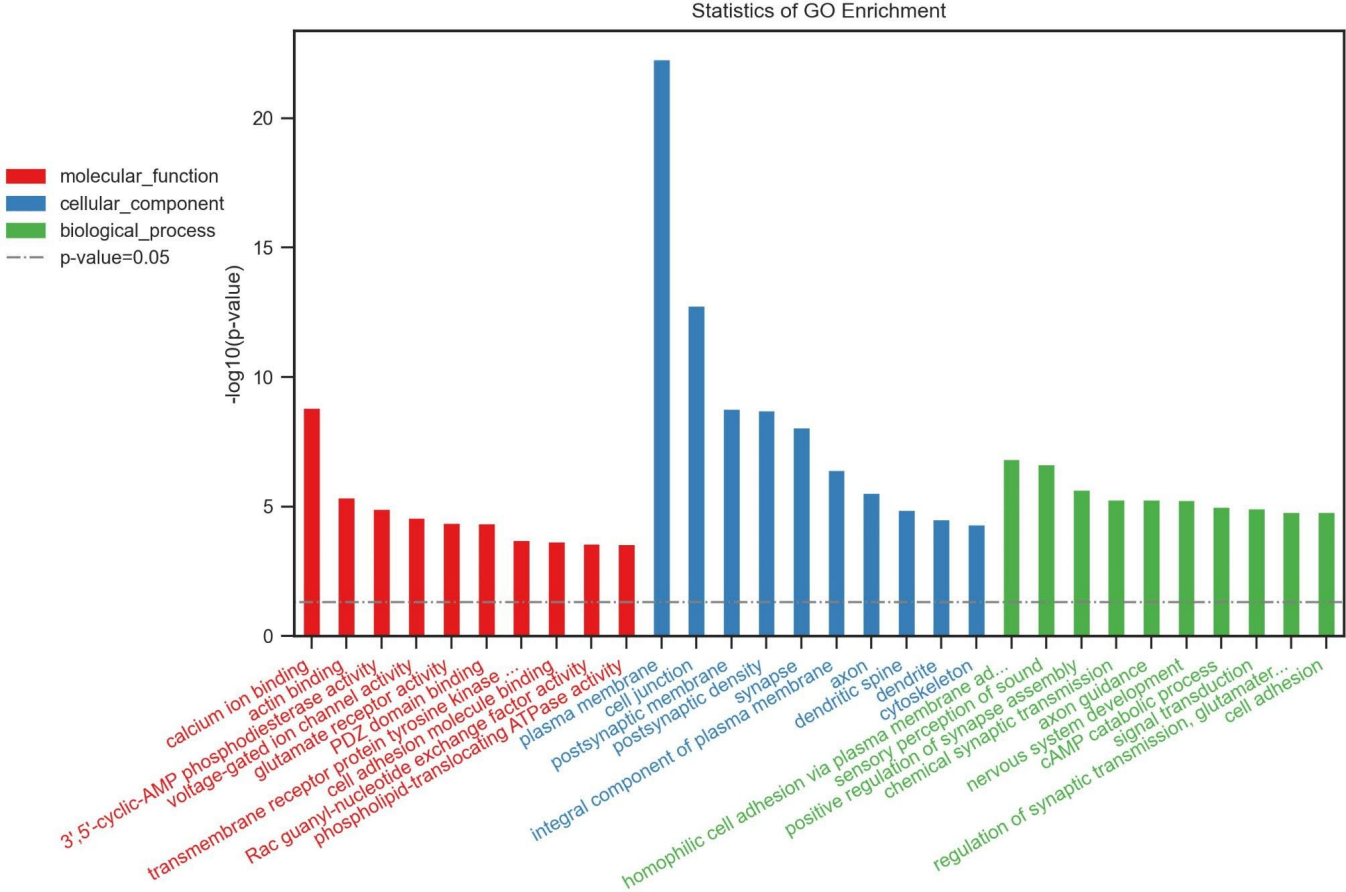
Statistics of Gene Ontology (GO) enrichment among all three Groups

Using the GO knowledgebase, we generated top 10 lists of molecular function, cell composition and biological process of differential target genes between Groups 1 and 2 (Fig. 1) and Groups 2 and 3 (Fig. 2) as well as among all three groups (Fig. 3). The top 10 lists of molecular function consisted of calcium ion binding, actin binding and 3ʹ,5ʹ-cyclic-AMP phosphodiesterase activity as well as voltage-gated ion channel activity, glutamate receptor activity, PDZ domain binding, transmembrane receptor protein tyrosine kinase, cell adhesion molecule binding, Rac guanyl-nucleotide exchange factor activity and phospholipid-translocating ATPase activity, whereas the top 10 lists of cell composition involved the GO terms plasma membrane, cell junction, postsynaptic membrane, postsynaptic density, synapse, integral component of plasma membrane, axon, dendritic spine, dendrite and cytoskeleton. Regarding biological process, the most common GO terms were homophilic cell adhesion via plasma membrane, sensory perception of sound, positive regulation of synapse assembly, chemical synaptic transmission, axon guidance, nervous system development, cAMP catabolic process, signal transduction, regulation of synaptic transmission and cell adhesion.

### KEGG signalling pathway analysis

**Fig. 4:**
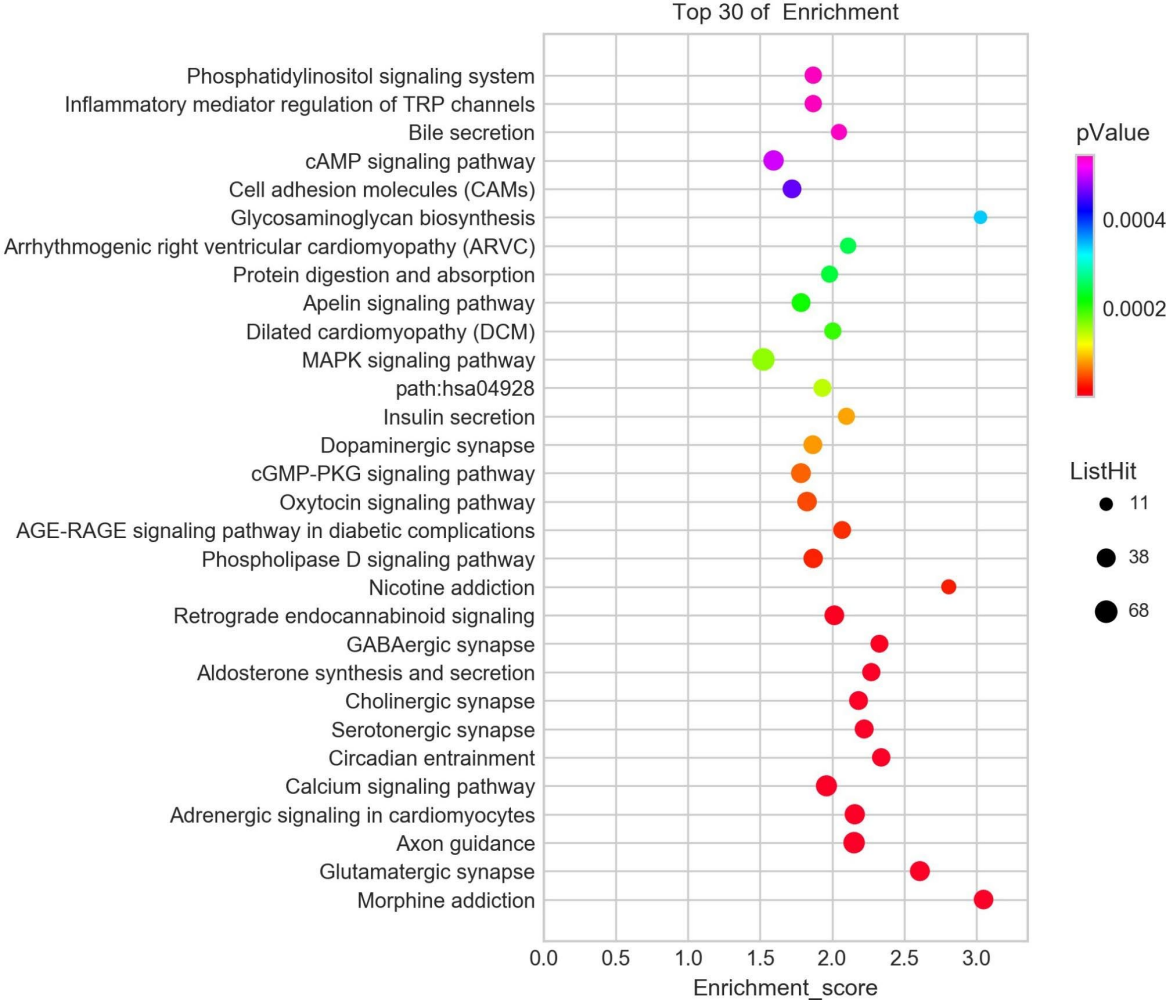
Statistics of Kyoto Encyclopedia of Genes and Genomes (KEGG) signal pathways between Groups 1 and 2

**Fig. 5:**
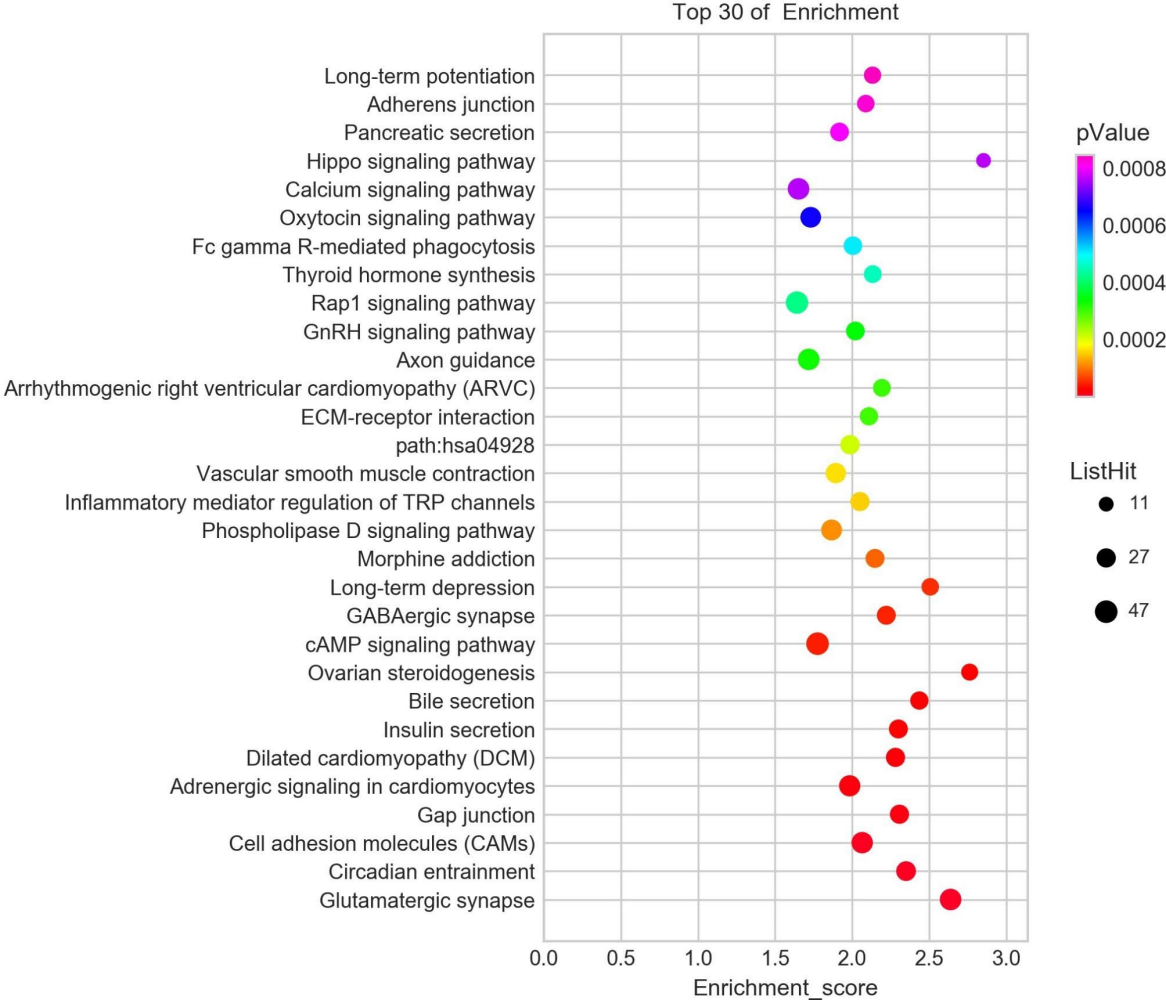
Statistics of Kyoto Encyclopedia of Genes and Genomes (KEGG) signal pathways between Groups 2 and 3

**Fig. 6:**
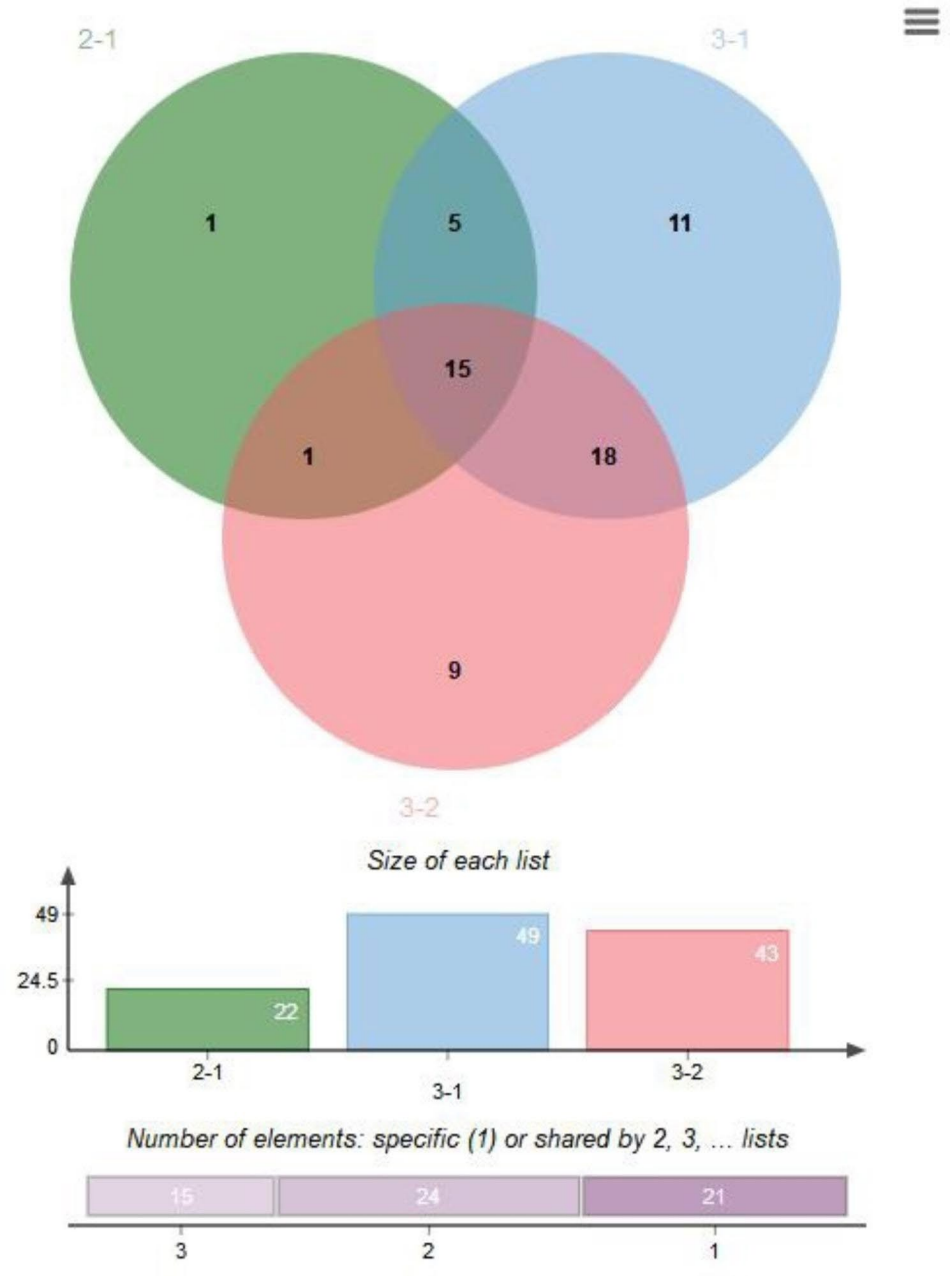
Intersections of Kyoto Encyclopedia of Genes and Genomes (KEGG) signal pathways among the three groups. “2 − 1”, “3 − 1” and “3 − 2” denote intersections of KEGG signal pathways between Groups 2 and 1, 3 and 1, and 3 and 2, respectively

The top 30 lists of differential target genes in KEGG signalling pathways between Groups 1 and 2 are shown in Fig. 4 and include the cAMP signalling pathway, cell adhesion molecules, mark signalling pathway, Apelin signalling pathway, divided cardiology pathway, aldosterone synthesis and secretion and the calcium signalling pathway, whereas those between Groups 2 and 3 include gap junction, cell adhesion molecules, adrenergic signalling in cardiomyocytes, DCM, cAMP signalling pathway, Hippo signalling pathway, calcium signalling pathway and inflammatory mediator regulation of TRP channels (Fig. 5). Among the three groups (Fig. 6), we identified differential target genes from the following 15 KEGG pathways: morphine addiction, glutamatergic synapse, axon guidance, adrenergic signalling in cardiomyocytes, serotonergic synapse, circadian entrainment, calcium signalling pathway, GABAergic synapse, aldosterone synthesis and secretion, oxytocin signalling pathway, phospholipase D signalling pathway, and insulin secretion as well as Apelin, DCM and MAPK signalling pathways. The detailed information on the differential target genes enriched in these KEGG signalling pathways is shown in Table 2.

**Table 2:** 15 KEGG signal pathways of differential target genes among three groups

No.	Pathways	Target genes	*p*-values
1	Morphine addiction	*ADCY8; GNGT2*	1.52 × 10^− 12^
2	Glutamatergic synapse	*ITPR1;ITPR2; ITPR3; ADCY8; PLCB1;GNG4*	1.71 × 10^− 10^
3	Axon guidance	*PIK3R6*	3.95 × 10^− 9^
4	Adrenergic signaling in cardiomyocytes	*ADCY7; ADCY8; SLC8A1; RYR2; PLCB1; PIK3R6*	8.68 × 10^− 8^
5	Serotonergic synapse	*ITPR1; ITPR2; PLCB1*	6.44 × 10^− 7^
6	Circadian entrainment	*ADCY8; ITPR1; RYR2; RYR3; PLCB1*	6.38 × 10^− 7^
7	Calcium signaling pathway	*ITPR1; ITPR2; ADCY8; SLC8A1; RYR2; RYR3; PLCB1*	3.41 × 10^− 7^
8	GABAergic synapse	*ADCY8; GNG2*	2.26 × 10^− 6^
9	Aldosterone synthesis and secretion	*ITPR1;ITPR2; ADCY8; PLCB1*	2.01 × 10^− 6^
10	Oxytocin signaling pathway	*ITPR1; ITPR2; PRKAG2; ADCY8; MEF2C; RYR2; RYR3; PLCB1*	4.59 × 10^− 5^
11	Phospholipase D signaling pathway	*ADCY8; PLCB1*	3.14 × 10^− 5^
12	Insulin secretion	*ADCY8; RYR2; PLCB1*	2.42 × 10^− 5^
13	Apelin signaling pathway	*ITPR1;ITPR2; PRKAG2; ADCY8; SLC8A1; MEF2C; RYR2; PLCB1; ATCA2; AGTR1*	2.13 × 10^− 4^
14	Dilated cardiomyopathy (DCM)	*ADCY8; SLC8A1; RYR2*	1.96 × 10^− 4^
15	MAPK signaling pathway	*MEF2C*	1.62 × 10^− 4^

**Abbreviations**: KEGG, Kyoto Encyclopedia of Genes and Genomes

### SNP analysis

Next, we analysed the significantly different SNP loci of the 15 KEGG pathways identified from the three patient groups, and the information on the most significant 26 SNPs, including chromosome number and gene and protein names, is shown in Table 3. These 26 SNPs are found in 17 genes as follows: five SNPs in *RYR2* and *RYR3*; five spanning *ITPR1*, *ITPR2* and *ITPR3*; four in *GNG2* and *GNG4*; two in *MEF2C* and *PRKAG2*; two in both *ADCY7* and *ADCY8* and a single SNP in *PIK3R6*, *ACTA2*, *GNGT2*, *SLC8A1*, *AGTR1* and *PLCB1*.

**Table 3:** Information of the significantly different SNP loci of the 15 KEGG pathways

SNP ID	Chr	Protein names	Gene names
rs12198325	6	Inositol 1,4,5-trisphosphate receptor	ITPR3
rs139061476	17	Phosphoinositide 3-kinase regulatory subunit 6	PIK3R6
rs142481236	12	Inositol 1,4,5-trisphosphate receptor type 2	ITPR2
rs1441734	10	Actin, aortic smooth muscle	ACTA2
rs2233362	17	Guanine nucleotide-binding protein G(I)/G(S)/G(O) subunit gamma-T2	GNGT2
rs58368463	7	5’-AMP-activated protein kinase subunit gamma-2	PRKAG2
rs10926318	1	Guanine nucleotide-binding protein G(I)/G(S)/G(O) subunit gamma-4	GNG4
rs12334868	8	Adenylate cyclase type 8	ADCY8
rs12439006	15	Ryanodine receptor 3	RYR3
rs12889199	14	Guanine nucleotide-binding protein G(I)/G(S)/G(O) subunit gamma-2	GNG2
rs16958069	15	Ryanodine receptor 3	RYR3
rs2296339	6	Inositol 1,4,5-trisphosphate receptor	ITPR3
rs2373792	2	Sodium/calcium exchanger 1	SLC8A1
rs28694719	14	Guanine nucleotide-binding protein G(I)/G(S)/G(O) subunit gamma-2	GNG2
rs3805018	3	Inositol 1,4,5-trisphosphate receptor type 1	ITPR1
rs4518438	5	Myocyte-specific enhancer factor 2 C	MEF2C
rs4785401	16	Adenylate cyclase type 7	ADCY7
rs57938337	1	Ryanodine receptor 2	RYR2
rs6683225	1	Ryanodine receptor 2	RYR2
rs6692782	1	Ryanodine receptor 2	RYR2
rs6801836	3	Type-1 angiotensin II receptor	AGTR1
rs7154813	14	Guanine nucleotide-binding protein G(I)/G(S)/G(O) subunit gamma-2	GNG2
rs770463	5	Myocyte-specific enhancer factor 2 C	MEF2C
rs80039831	7	5’-AMP-activated protein kinase subunit gamma-2	PRKAG2
rs8114499	20	1-phosphatidylinositol 4,5-bisphosphate phosphodiesterase beta-1	PLCB1
rs931388	3	Inositol 1,4,5-trisphosphate receptor type 1	ITPR1

Abbreviations: SNP, single nucleotide polymorphism; KEGG, Kyoto Encyclopedia of Genes and Genomes; Chr, chromosome

The genotype and allele frequencies of the five SNPs in *RYR2* (rs57938337, rs6683225 and rs6692782) and *RYR3* (rs12439006 and rs16958069) were then analysed as shown in Table 4. We found that the proportion of the GG genotype and G allele frequency of rs57938337 in *RYR2* of patients from both Groups 2 and 3 (Group 2 + 3) were higher than those in Group 1, whereas proportions of the GG genotype and frequencies of the G allele of rs6683225 and rs6692782 were significantly lower than those in Group 1. In addition, compared with Group 1, we found that the proportion of the AA genotype and A allele frequency of rs12439006 in *RYR3* of pooled patients (Group 2 + 3) were significantly lower, whereas those of GA/GG genotypes and G allele frequency were higher. Similarly, the proportion of the AA genotype and frequency of the A allele of rs16958069 in pooled patients were lower than those in Group 1, whereas proportions of the AC/CC genotypes and C allele frequency in pooled patients (Group 2 + 3) were higher than those in patients from Group 1.

**Table 4:** Analysis of the genotype and allele frequency of SNPs on RYR2 and RYR3

	Group1	Group2 + 3	Chi-square	P value
	N = 26	N = 73		
**RYR2**				
**rs57938337**				
GG	11(42.3)	48(65.8)	4.377	0.0364
AA + GA	15(57.7)	25(34.2)		
G	33(63.5)	120(82.2)	7.660	0.0056
A	19(36.5)	26(17.8)		
**rs6683225**				
GG	19(73.1)	34(46.6)	5.413	0.02
AA + GA	7(26.9)	39(53.4)		
G	45(86.5)	97(66.4)	7.637	0.0057
A	7(13.5)	49(33.6)		
**rs6692782**				
GG	14(53.8)	22(30.1)	4.657	0.0309
AA + GA	12(46.2)	51(69.9)		
G	40(76.9)	80(54.8)	7.864	0.0050
A	12(23.1)	66(45.2)		
**RYR3**				
**rs12439006**				
AA	21(80.8)	33(45.2)	9.780	0.0018
GA + GG	5(19.2)	40(54.8)		
A	46(88.5)	44(30.1)	7.027	0.0080
G	6(11.5)	102(69.9)		
**rs16958069**				
AA	15(57.7)	25(34.2)	4.377	0.0364
AC + CC	11(42.3)	48(65.8)		
A	41(78.8)	58(39.7)	5.825	0.0158
C	11(21.2)	88(60.3)		

**Abbreviations**: SNP, single nucleotide polymorphism; RYR, Ryanodine receptor

## Discussion

Over-activation of the neuro-humoral system, including the RAAS and SNS, are important pathophysiological mechanisms for the occurrence and development of cardiorenal syndrome [18]. Recent studies have shown that genetic polymorphisms are a “hot spot” in the aetiological mechanism of HF; variations in genes encoding the β adrenoceptor and norepinephrine transporter are associated with the occurrence and development of HF [19–22]. However, it remains unclear whether genetic susceptibility plays a role in the onset and progression of CRS.

In this study, we used a bioinformatics approach to analyse patients with DCM-induced HF and different stages of RI, ranging from none to severe, and identified significant differences among our three subgroups in target genes of different signalling pathways, including the myocardial adrenergic signalling pathway, aldosterone synthesis and secretion pathway as well as the DCM and calcium ion signalling pathways. These pathways are related to underlying pathophysiological mechanisms in HF, such as SNS and RAAS activation. Importantly, the patients in this study were definitively diagnosed with DCM based on the current gold standard. DCM is a type of heart disease that is characterized by left or right ventricular dilation, which is associated with reduced systolic function, thereby leading to irreversible HF. It is the most common non-ischemic cardiomyopathy worldwide, with an annual incidence rate of about 7 cases per 100,000 population [23, 24].

Recent studies have found that the mechanism underlying DCM is related to mutations in genes encoding cytoskeletal, contraction or inner nuclear membrane proteins [25, 26]. The 26 SNPs of the significantly enriched target genes identified in this study are mainly found in genes encoding ion channels or transporters involved in the release and transport of calcium ions in the cytoplasm or plasma membrane, whereas others encode either myocardial skeletal proteins or myocardial-specific transcription factors.

Further, of these 26 SNPs, three SNPs (rs57938337, rs6683225 and rs6692782) were located in *RYR2* and two SNPs (rs12439006 and rs16958069) were located in *RYR3*, both of which encode ryanodine receptors. These receptors are located in the sarcoplasmic/endoplasmic reticulum. The plasma membrane and sarcoplasmic reticulum are responsible for the release of intracellular calcium and the excitation–contraction coupling of myocardium and skeletal muscle. Currently, there are three known isomers, namely, RYR1, RYR2 and RYR3. RYR1 and RYR2 are highly expressed in skeletal muscle and myocardium, respectively; RYR3 is not only expressed in skeletal muscle, but also widely expressed in hippocampal neurons, thalamus, vascular smooth muscle cells, lung, kidney, aorta and other tissues and organs of the vascular system [27].

Shrestha et al. previously used whole-genome sequencing to study 177 male Caucasian patients positive for HIV and found that two SNPs, rs2229116 and rs7177922, in *RYR3* were associated with carotid intimal thickness [28]. In addition, studies on different Han Chinese populations showed that three SNPs, rs2033610, rs2596164 and rs2278317, in *RYR3* were associated with the incidence of hypertension, diabetes and Alzheimer’s disease [29]. Further, Galati et al. studied 235 patients implanted with defibrillators and found that a SNP at Q2958R in RYR2 was significantly correlated with malignant arrhythmia [30]. These findings demonstrate that multiple SNPs in *RYR2* and *RYR3* are associated with either cardiovascular disease or cardiovascular risk factors.

The calcium ion is an important secondary messenger, and once activated, it directly binds to cardiac or skeletal troponin to cause muscle contraction. It also causes a protein phosphorylation cascade by activating protein kinase, thereby leading to various cellular effects. Under normal physiological conditions, the free calcium concentration in extracellular fluid is much higher than that of the intracellular calcium concentration, and while more than 90% of intracellular calcium ions are stored in the endoplasmic reticulum and mitochondria, the calcium concentration in cytoplasm is very low. If calcium channels on the plasma membrane or intracellular calcium pool are opened, it may lead to an influx of extracellular calcium or the release of calcium from the intracellular calcium pool, resulting in a sharp increase in the concentration of intracellular calcium [31]. In this study, using both GO enrichment and KEGG signalling pathway analysis, we identified calcium binding-related genes and the calcium signalling pathway in patients with HF and RI. In myocardium and skeletal muscle, ryanodine receptors are important calcium release channels in the endoplasmic reticulum and sarcoplasmic reticulum, mainly releasing calcium ions through calcium-induced calcium release (CICR) or depolarization-induced calcium release [27]. According to previous research, mutations in the cardiac sodium channel Na_v_1.5 gene (*SCN5A*) are associated with DCM, which is evidence of sodium channel involvement in the pathogenesis of DCM. However, pathways that cause ventricular dilatation and dysfunction associated with *SCN5A* mutations remain unclear [32, 33]. Whether other ion channel pathways are involved in the pathogenesis of RI in patients with DCM-induced HF remains to be further observed in large-scale clinical studies.

Recently, the important role of renal microcirculation in renal pathophysiology has attracted much attention. Renal microcirculation plays a key role in urinary sodium excretion and blood pressure control. The calcium signal of the anterior glomerular artery and renal corpuscle are the main determinants of pre-glomerular vascular resistance and are involved in regulating renal blood flow, glomerular capillary pressure and filtration rate (GFR), cortical peritubular perfusion and renal medullary blood flow [34]. Studies have shown that renal ryanodine receptors and CICR not only regulate the relaxation and contraction of renal vessels but also regulate the synthesis and release of renin by juxtaglomerular cells and the transport of water and sodium by renal tubules [35, 36]. These studies suggest that ryanodine receptors are involved in the pathophysiological mechanism of cardiorenal syndrome. We found that the frequencies of the rs12439006 G allele and rs16958069 C allele in *RYR3* were significantly higher in patients with HF and cardiorenal syndrome than those with HF alone. Owing to our modest sample size, whether these two SNPs are related to cardiorenal syndrome require confirmation by larger, multi-centre validation studies. In addition, whether these SNPs lead to functional changes in *RYR3* and affect renal microcirculation remain to be further studied.

A variant in the CLCNKA gene (SNP rs10927887; p.Arg83Gly) previously linked to HF was found to be associated with the eGFR and could contribute to explaining the risk of developing HF in Caucasian populations [37]. Recently, a prospective cohort study in a Japanese population found a significant association between the homozygous A-allele of rs12058375 and the presence of CRS. Kaplan Meier analysis demonstrated that homozygous A-allele carriers of rs12058375 had the greatest risk of developing cardiovascular events among the NPHP4 variants [38]. The above rs10927887 or rs12058375 was not included in the 26 SNPs of the significantly enriched target genes identified in our study, which may be because of race or sample size differences.

Several limitations of our study warrant discussion. First, the sample size is relatively small. Second, there is no follow-up observation on the correlation between SNPs loci and adverse cardiovascular events. Third, there is a lack of in vitro experiments on loss- and gain-of-function among these variants.

## Conclusions

Twenty-six significantly different SNP loci in 17 genes of the 15 KEGG pathways were found in the three patient groups. Among these variants, rs57938337, rs6683225 and rs6692782 in RYR2 and rs12439006 and rs16958069 in RYR3 are associated with RI in Han Chinese patients with heart failure, suggesting that these variants may be used to identify patients susceptible to CRS in the future. A larger sample size is needed in the future to further confirm the association between these SNPs and phenotypes in the Chinese population.

## Data Availability

The datasets used and/or analysed during the current study available from the corresponding author on reasonable request.
